# Continuous Fabrication of Ti_3_C_2_T_*x*_ MXene-Based Braided Coaxial Zinc-Ion Hybrid Supercapacitors with Improved Performance

**DOI:** 10.1007/s40820-021-00757-6

**Published:** 2021-12-15

**Authors:** Bao Shi, La Li, Aibing Chen, Tien-Chien Jen, Xinying Liu, Guozhen Shen

**Affiliations:** 1 Hebei University of Science and Technology, 70 Yuhua Road, Shijiazhuang, 050018 People’s Republic of China; 2 State Key Laboratory for Superlattices and Microstructures Institute of Semiconductors Chinese Academy of Sciences & Center of Materials Science and Optoelectronic Engineering, University of Chinese Academy of Sciences, Beijing, 100083 People’s Republic of China; 3 Department of Mechanical Engineering Science, Kingsway Campus, University of Johannesburg, Auckland Park, Johannesburg, 2006 South Africa; 4 Institute for Development of Energy for African Sustainability, University of South Africa, Private Bag X6, Florida, 1710 South Africa

**Keywords:** Ti_3_C_2_T_*x*_, MXene, Fiber supercapacitor, Coaxial structure, Zinc-ion

## Abstract

**Highlights:**

- Ti_3_C_2_T_*x*_ MXene-based coaxial zinc-ion hybrid fiber supercapacitors (FSCs) were fabricated with braided structure, which can be prepared continuously and present excellent flexibility and ultrastability.
- A sports watch driven by the watch belts which weaved uses the obtained zinc-ion hybrid FSC and LED arrays lighted by the FSCs under embedding into textiles, demonstrating the great potential application in smart wearable textiles.

**Abstract:**

Zinc-ion hybrid fiber supercapacitors (FSCs) are promising energy storages for wearable electronics owing to their high energy density, good flexibility, and weavability. However, it is still a critical challenge to optimize the structure of the designed FSC to improve energy density and realize the continuous fabrication of super-long FSCs. Herein, we propose a braided coaxial zinc-ion hybrid FSC with several meters of Ti_3_C_2_T_*x*_ MXene cathode as core electrodes, and shell zinc fiber anode was braided on the surface of the Ti_3_C_2_T_*x*_ MXene fibers across the solid electrolytes. According to the simulated results using ANSYS Maxwell software, the braided structures revealed a higher capacitance compared to the spring-like structures. The resulting FSCs exhibited a high areal capacitance of 214 mF cm^–2^, the energy density of 42.8 μWh cm^−2^ at 5 mV s^−1^, and excellent cycling stability with 83.58% capacity retention after 5000 cycles. The coaxial FSC was tied several kinds of knots, proving a shape-controllable fiber energy storage. Furthermore, the knitted FSC showed superior stability and weavability, which can be woven into watch belts or embedded into textiles to power smart watches and LED arrays for a few days.
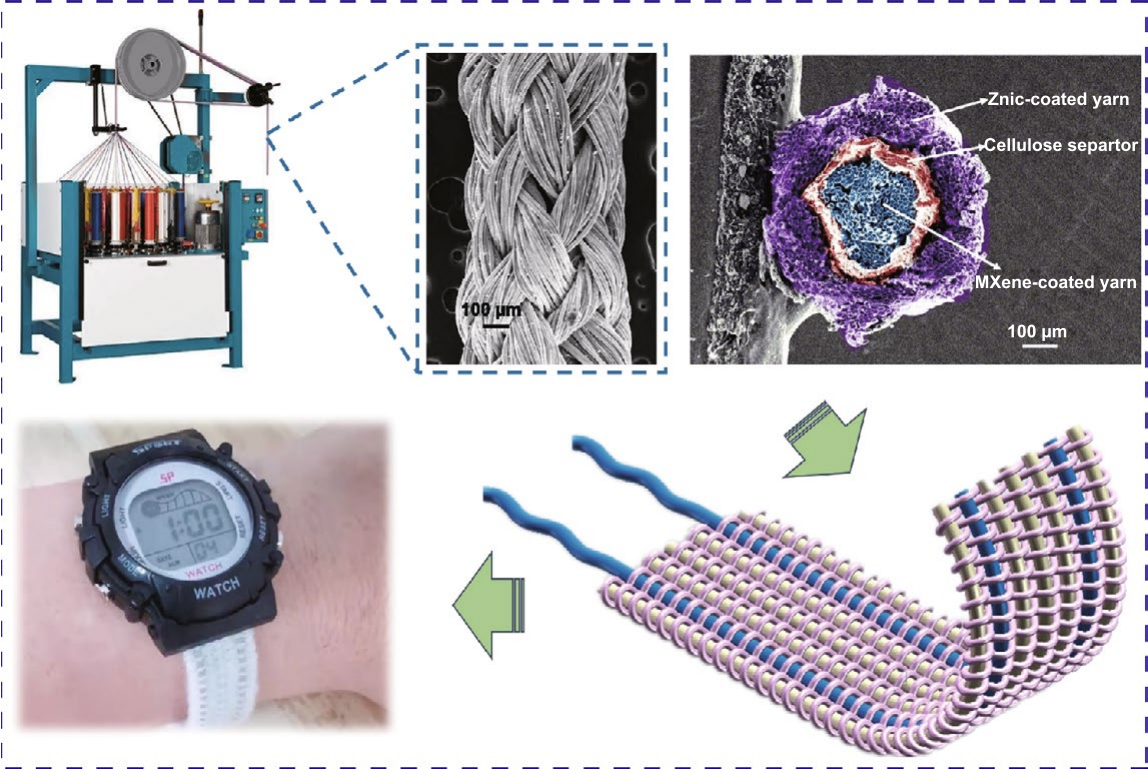

**Supplementary Information:**

The online version contains supplementary material available at 10.1007/s40820-021-00757-6.

## Introduction

Fiber electronics is a newly emerged flexible device developed in recent years, which offers unique ultra-flexible, shape adaptable, and weavability properties, allowing various deformations include bending, stretching, twisting, etc. [1, 2]. Up to date, different kinds of fiber devices such as fiber generators, sensors, detectors have been reported [3–5]. These fiber devices have increasingly seeking for the matched fiber energy storage to realize the stable, independent, and complete application circle [6]. The typical design of the fiber energy storage consists of fiber Li-ion battery, supercapacitors (SCs), hybrid supercapacitors [7]. Fiber hybrid supercapacitor (FSC) that employs metal-ion as anode with high capacitance, ultra-long serving time, and robust security is well developed due to its wide applications in wearable electronics, smart clothes, and multi-functional integrated textiles [8]. It is found the structures of the FSC have a profound influence on electrochemical behaviors. For example, Peng’s group reported the specific capacitance of coaxial SCs exhibited approximately tenfold higher than that of twisted ones [9]. Therefore, the FSC with novel coaxial structures needs further to be developed to meet the practical demands of fiber electronic devices. As an important branch of the hybrid FSC, zinc-ion-based FSCs that possess superior characteristics, such as high theoretical capacity (820 mAh g^−1^ and 5855 mAh cm^−3^), high safety, nontoxicity, low cost, abundant resources (about 300 times higher than lithium), and lightweight, are considered as an ideal energy supply unit [10]. Although research on the Zn-ion hybrid SCs has been expanding rapidly, the Zn-ion-based FSC is relatively seldom explored.

Carbides and nitrides (MXenes) with two-dimensional (2D) structures have been demonstrated as promising cathode materials for the fabrication of Zn-ion hybrid SC due to their high electrical conductivity (up to 20,000 S cm^−1^) and excellent specific capacitance (up to 1500 F cm^−3^) [11–14]. Moreover, different from traditional hydrophobic carbon materials like graphene, carbon nanotube, MXenes contain abundant surface functional groups offer hydrophilic properties, which are suitable for solution processing and fiber device fabrication by spray coating, printing, and coating, etc. [15–24]. Unfortunately, the Ti_3_C_2_T_*x*_ Mxene-based Zn-ion FSC has rarely been reported in the previous literature; the reason and difficulty in the fabrication of Zn//Ti_3_C_2_T_*x*_ MXene FSC are summarized in the following three points. 1) The preparation of the super-long electrodes, ensuring continuous fabrication of FSC. 2) The optimization of the structures of the FSC for enhancing the electrochemical performance of the assembled devices. 3) The selection of the solid-state electrolyte, which endows the FSC with stable and wearable properties. To solve the problems mentioned above, first of all, the dynamic dipping and electrodeposition equipment for preparing super-long Ti_3_C_2_T_*x*_ fiber cathode and Zn anode need to be designed [25]. Secondly, FSC is usually divided into two main types, including twisted and coaxial structures [26–28]. The coaxial structure has been demonstrated better performances compared to twisted. Coaxial FSCs can be further broken up into two forms consisting of braided and winded. Braided shell electrodes with parallel crossed network structures have smaller resistance, easier to collect and transfer charge, which is expected a better performance. Moreover, the braided coaxial FSCs can weave fibers into continuous tubular structures, realizing a continuous super-long fabrication of the coaxial FSC [29, 30]. Finally, the novel all-solid-state electrolyte needs to be synthesized.

Taking all these into account, we provide a facile approach to fabricate a superlong coaxial FSC with Ti_3_C_2_T_*x*_ MXene fibers core cathode and Zinc fibers shell anode, which was braided into the tubular structure and wrapped around the cathode. The fabricated coaxial FSC showed high areal capacitance (up to 214 mF cm^−2^), energy density (42.8 μWh cm^−2^) at 5 mV s^−1^, and excellent cycling stability of 83.58% capacity retention after 5000 cycles. Moreover, the simulated results using ANSYS Maxwell software also exhibited higher capacitance of braided coaxial FSC. In order to prove the weavability and high energy density, the coaxial FSC was woven into textiles and used to power LED lights and smart watches.

## Experimental Section

### Materials

Silver-plated nylon fibers were purchased from Qingdao Tianyin Textile Technology Co., Ltd. Ti_3_AlC_2_ was purchased from Carbon-Ukraine. Lithium fluoride (LiF), hydrochloric acid (HCl), zinc oxide (ZnO), sodium hydroxide (NaOH), zinc sulfate monohydrate (ZnSO_4_·H_2_O), gelatin were obtained from Shanghai Aladdin Biochemical Technology Co., Ltd. Cellulose diaphragm was provided by Tianjin Annuohe New Energy Technology Co., Ltd.

### Preparation of 2D Delaminated Ti_3_C_2_T_x_ MXene

Ti_3_C_2_T_*x*_ MXene nanoplates were prepared according to the previously reported selective etching route. Typically, 1 g of Ti_3_AlC_2_ powder was slowly added into the mixture solution of 1 g LiF and 20 mL HCl (9 M) and then magnetically stirred at 35 ℃ for 24 h. The resulting suspension was washed with deionized (DI) water and centrifuged several times until the pH value of the solution reached neutral to obtain multi-layer Ti_3_C_2_T_*x*_ MXene. Subsequently, the solution was sonicated in the ice bath for 1 h. Finally, the Ti_3_C_2_T_*x*_ solution was collected by centrifuging at 3500 rpm for 5 min, while small-sized Ti_3_C_2_T_*x*_ MXene was obtained.

### Preparation of Solid-state Electrolyte

Solid-state electrolyte was prepared by adding appropriate 4.5 g gelatin into 30 mL ZnSO_4_ (1.5 M); then, the solution was magnetically stirred at 60 ℃ for 30 min to obtain a clear solution. Finally, the solution was naturally cooled and solidified at room temperature.

## Results and Discussion

### Material Characterizations

**Fig. 1:**
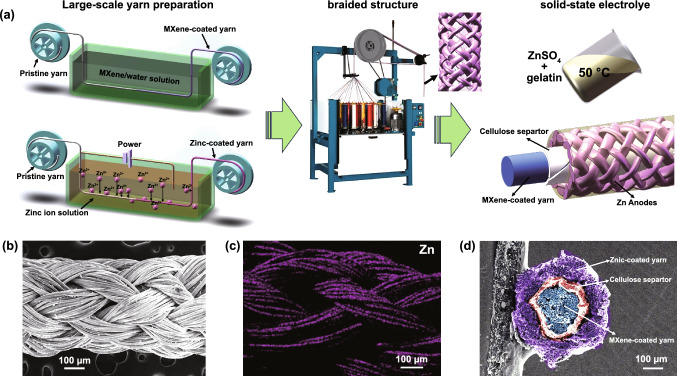
Schematic diagram of the braided coaxial FSC. **a** Schematic illustration of the large-scale production of MXene and zinc-coated fibers and braided coaxial FSC. **b** SEM image of FSC. **c** Mapping image of zinc element on the surface of FSC. **d** Cross-sectional SEM image of a coaxial FSC

Figure 1a shows the continuous fiber manufacturing process to assemble Zn-/MXene-based braided coaxial FSC. The homemade semi-automatic equipment was employed to prepare Ti_3_C_2_T_*x*_ cathode by the dynamic multiple dipping fiber into MXene suspension. The concentration of the Ti_3_C_2_T_*x*_ suspension was about 10 mg mL^**−1**^. After drying the Ti_3_C_2_T_*x*_ fiber cathode, the mass loading of 0.15 mg cm^**−1**^ for the Ti_3_C_2_T_*x*_ cathode was obtained. The length of the prepared Ti_3_C_2_T_*x*_ MXene fiber cathode reached up to 1.5 m, as shown in Fig. S1a. The Zn fiber anode (Fig. S1b) was made by the continuous electrodeposition method. The corresponding homemade electrodeposition equipment is displayed in Fig. S1c. In a typical three-electrode electrochemical workstation system, two pieces of Zinc plate paralleled with the fiber served as work electrode and Hg/HgO worked as the reference electrode; the pre-washed silver-plated fibers (100 D 36 f) were connected to the counterelectrode and went through the electrolyte that contains a mixture of 0.1 M zinc oxide (ZnO) and 3.75 M sodium hydroxide (NaOH) at a different speed. The length of the silver-plated fibers immersion in the electrolyte is 12 cm. The constant current used in the electrodeposition process was tuned from 6 to 9 mA. After the electrodeposition process, the Zn coated fiber was immediately washed in DI water and dried at 40 ℃ to prevent oxidation. The SEM images of the Zn fiber anode at different currents and move speeds are provided in Fig. S4. From the SEM images, it is found the Zn on the surface of the fiber exhibited uniform formation and a suitable amount when the moving speed of the fiber was 2 cm min^−1^ at the constant current of 7 mA. Next, a cellulose diaphragm of 35 μm thick was winded on the surface of the Ti_3_C_2_T_*x*_ MXene-coated fiber. Zinc-coated fiber anodes were then woven into a network layer of tubular fabric with plain texture on the surface of the cellulose diaphragm by a 2D knitting machine. Six yarns were woven clockwise and the other six anticlockwise around the MXene cathode, intersecting in pairs to form the outer layer of three-layer coaxial FSC. Finally, the solid electrolyte made of 1.5 M ZnSO_4_ and 15.0 W/V % of gelatin was added. The braided FSC was fully immersed in a gelatin electrolyte and kept at 50 ℃ for 10 min to prepare a fully solid-state FSC. The SEM image of the fabricated coaxial FSC is presented in Fig. 1b. The braided structure of the Zn anode can be clearly seen. Figure 1c depicts the mapping images of the coaxial FSC structure, which proves that zinc elements are evenly distributed throughout the surface of the fibers. Figure S2 depicts the SEM and mapping images of the Ti_3_C_2_T_*x*_ MXene cathode. The cross-sectional SEM image of the coaxial FSC (Fig. 1d) shows that the FSC consists of shell-like Zn anode, cellulose membrane, and the core Ti_3_C_2_T_*x*_ MXene cathode, providing a separated, uniform, stable, and continuous structure.

### Comparison of Braided and Winded Coaxial FSCs

**Fig. 2:**
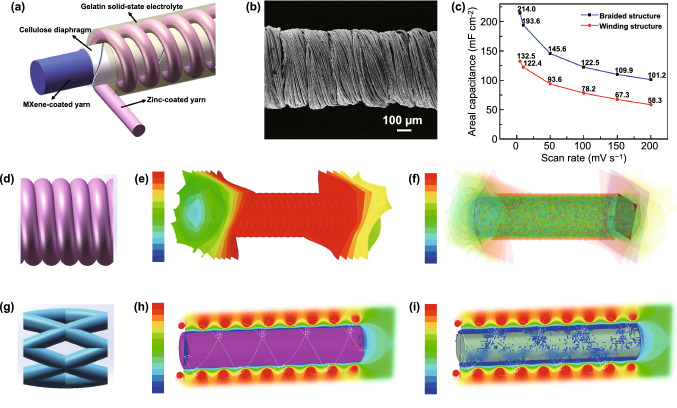
**a** Structural diagram of winded coaxial FSC. **b** SEM image of winded coaxial FSC. **c** Comparison of area capacitance of braided and winded coaxial FSCs. **d, g** 3D models of two kinds of coaxial FSCs. **e, h** Simulated voltage distribution of braided and winded coaxial FSCs. **f, i** Simulated energy distribution of braided and winded coaxial FSCs

To verify the better electrochemical performance of the coaxial FSC with braided structures, the contrast winded coaxial FSC was also fabricated, as shown in Fig. 2a, b. The serried spring-like Zn cathode can be noticed in the SEM images (Fig. 2b). Figure 2c demonstrates that braided FSCs delivered higher specific areal capacitances of 214 mF cm^**−**2^, which is almost twice that of winded FSCs at scan rates of 5 mV s^**−1**^. To uncover the reason for the better performance of the braided FSC, the potential and energy distributions simulated by the ANSYS Maxwell analysis software of the two kinds of FSCs were presented. The corresponding parameters are provided (Table S1). Figure 2d, g exhibits the 3D models of braided and winded coaxial FSCs according to the simulating parameters, respectively. Next, the material properties of the models and electrolyte parameters were defined in ANSYS Maxwell analysis software; a voltage of − 0.6 to 0.6 V was added between the two electrodes. Finally, solution settings were added to the analysis voltage field and energy field for simulation analysis.

Figure 2e, h depicts the potential distributions between anode and cathode when a voltage ranging from − 0.6 to 0.6 V was applied to the devices, respectively. The negative and positive voltage used in the analysis process was potential difference related to the electroneutral electrolyte. In addition, large span voltage could provide more clearly charge distribution images. Compared with winded FSCs, braided coaxial FSCs possess a much more uniform potential distribution and higher electric charge-transfer effect in the axis direction since the external fibers of braided coaxial FSC have a parallel form leading to faster charge collection and transfer rates, while in winded coaxial FSC, the charge transfer within a prolonged path increases the resistance, which is not beneficial for the electrochemical performance. Figure 2f, i presents the energy distributions in both structural FSCs. From the distribution images, we can see the braided devices have four electrode branches knitted in parallel, providing an increased energy density. Such a braided structure also maintains the charge transfer function in case of one or two fiber electrodes is cut or broken. In contrast, the winded one with a single shell electrode is disrupted when anywhere from the shell electrode is victimized, disabling the charge storage and transfer functions. The theoretical specific capacitance was calculated according to the corresponding calculation formula [30]. A capacitance ratio of 1.53:1 was obtained, which is consistent with the experimental results, confirming and explaining the better electrochemical performances of the braided coaxial FSC.

### Electrochemical Performance Analysis

**Fig. 3:**
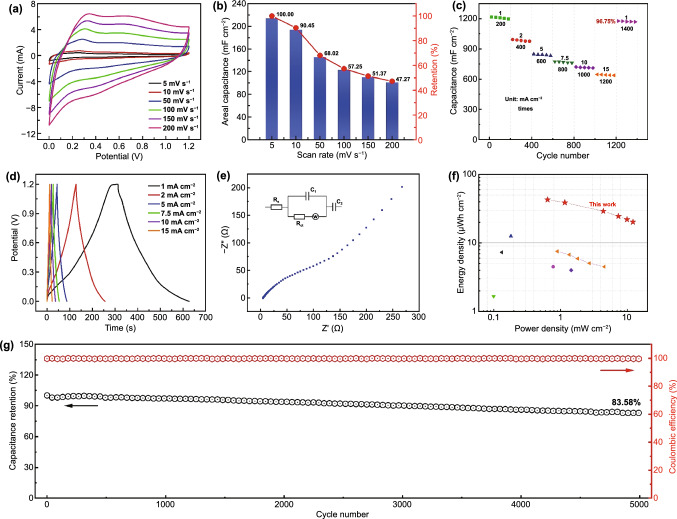
Electrochemical performance of the braided coaxial FSC. **a** CV curves of 2 cm of the braided coaxial FSC at various scan rates. **b** Areal capacitance with respect to scan rates. **c** Areal capacitance with respect of cycle number at different current densities. **d** Galvanostatic charge–discharge curves at different current densities. **e** EIS curve of the FSC. **f** Comparisons of the energy and power densities with previously reported works. **g** Cycling performance and the corresponding coulombic efficiency of the fabricated FSC at 20 mA cm^−2^

The electrochemical properties of the braided coaxial Zn//Ti_3_C_2_T_*x*_ FSC with the length of 2 cm were systematically gathered, as shown in Fig. 3. Figure 3a displays the CV curves of the braided coaxial FSC in a voltage window of 1.2 V at scan rates ranging from 5 to 200 mV s^**−1**^, respectively. Obvious redox peaks at the lower potential range can be observed, which are caused by the insertion of Zn ion into the Ti_3_C_2_T_*x*_ layers, revealing the combined energy storage mechanism of pseudoredox reaction and double-layer effect. In detail, when charging the Zn//Ti_3_C_2_T_*x*_ fiber devices, Zn transforms to Zn^2+^ and moves from anode to cathode, then intercalates into the Ti_3_C_2_T_*x*_ layers, or adsorbs on the surface of the Ti_3_C_2_T_*x*_ cathode. When the device charges, the procedure is the inverse of the above process. With the increase in scanning rate, the shape of the CV curves was unchanged, indicating that the coaxial FSC has a remarkable rate capability and excellent capacitive behavior. Figure 3b shows the calculated specific area capacitances of 214.00 to 101.15 mF cm^**−**2^ at increasing scanning rates from 5 to 100 mV s^**−1**^, with the coulombic efficiency of 100%, respectively. Almost 50% capacitance remained as the scan rate increased 20 times, demonstrating the superior rate stability of the fabricated devices. Figure 3c shows the areal capacitance with respect to cycle number at different current densities. It can be seen the 96.75% of the initial capacitance remained after 1600 cycles. The galvanostatic charge–discharge (GCD) curves of the designed FSC (Fig. 3d) with a stable voltage window of 0–1.2 V show the symmetric triangular shapes at higher current densities (> 5 mA cm^**−1**^) and slight deviation at current density of 1 mA cm^**−**2^, revealing the capacitive and pseudoredox reaction charge storage behavior. Electrochemical impedance spectroscopy (EIS) was conducted to understand the charge transfer and ion transport properties of the coaxial FSC. As shown in Fig. 3e, the Nyquist curve obtained in frequencies ranging from 100 kHz to 0.01 Hz confirmed the low charge transport resistance of 4.41 Ω. Figure 3f displays the compared energy and power densities of this FSC device and reported works; the highest energy density of 42.8 μWh cm^**−**2^ at a power density of 0.64 mW cm^**−**2^ of our devices can be calculated, which is much higher than that of recently reported works as shown in Table 1 [31–40]. Figure 3g exhibits the capacity retention of the braided coaxial FSC after 5000 charge/discharge cycles at a current density of 20 mA cm^**−**2^; 83.6% of initial value was remained with high Coulombic efficiency of about 100%, suggesting the outstanding cycling stabilities of the fabricated Zn//Ti_3_C_2_T_*x*_ FSC.

**Table 1:** A review of the fiber supercapacitors with different electrode materials

Electrodes	Power density (mW cm^−2^)	Energy density (μWh cm^−2^)	Refs.
Ti_3_C_2_T_*x*_ MXene-coated activated carbon cloth	0.779	4.5	[31]
Ti_3_C_2_T_*x*_ MXene-coated silk-derived carbon cloth	0.18099	12.5694	[32]
Ti_3_C_2_T_*x*_ MXene-coated metal mesh	0.0999	1.665	[33]
Ti_3_C_2_T_*x*_ MXene-coated nylon	1.44108	4.003	[34]
Ti_3_C_2_T_*x*_ MXene-coated silver-plated nylon fibers	0.132	7.3	[35]
Zinc and Ti_3_C_2_T_*x*_ MXene	0.9	7.5375	[36]
Carbon nanotube / MnO_2_-3	0.06	15.2	[37]
Mn/Mo@multiwalled carbon nanotube	1	4.2	[38]
Graphene / CNT	1.22	1.27	[39]
Multiwalled carbon nanotube / Ppy	0.1765	0.44	[40]
This work	12.138	20.23	–

### Flexibility of Fabricated FSC

**Fig. 4:**
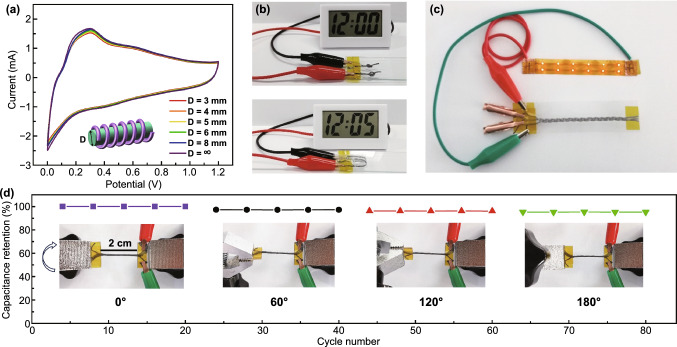
Bending, knotting, and twisting properties of fabricated FSC. **a** CV curves of coaxial FSC under bending (labeled with ∞) and bent with different curvatures diameter D. **b** Digital photographs of the FSCs with 2 cm powering electronic clocks at bending and knotting state. **c** A 12 LED array lightened by three SCs connected in series. **d** Capacitance retentions after twisting at increasing degrees up to 80 cycles

The flexibility and weavability of the fabricated Zn//Ti_3_C_2_T_*x*_ FSC were then measured. The CV curves of the coaxial FSC bent with different curvature diameters are compared in Fig. 4a. The shapes of the CV curves are maintained, showing that the FSC has excellent flexibility, which is vital for wearable energy storage devices. In order to demonstrate the superior stability of coaxial FSC, two FSCs with a length of 3 cm were connected in series to power an electronic clock, as shown in Fig. 4b. Coaxial FSCs can normally and stably power the electronic timepiece under knotting and bending, and the power supply duration was able to reach 110 min (Fig. S5). Moreover, 12 red LED lights could be lighted by 3 FSCs connected in series. It is worth noting that the 3 FSCs were easily knitted in a textile (Fig. 4c). The coaxial FSC may twisted during the weaving process and practical application, so we also tested the twisted performance of coaxial FSC. The capacitance of the coaxial FSC with one end fixed and the other end rotated at different angles was measured, as shown in Fig. 4d. The capacitance is maintained 95% of the original value when the FSC rotated by 180 degrees, proving the good flexibility of the devices.

### Application of Coaxial FSCs

**Fig. 5:**
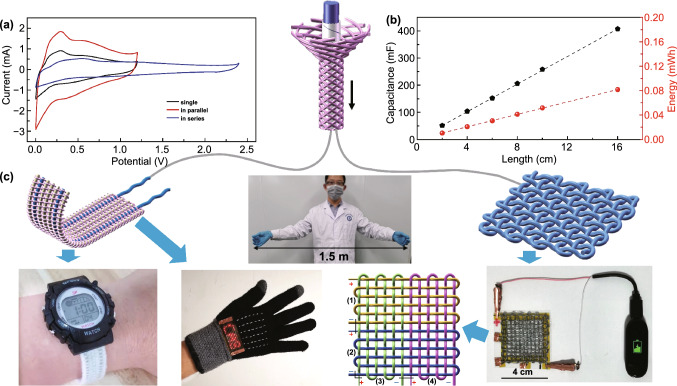
Application of coaxial FSCs in textiles. **a** CV curves of a single FSC and the two FSCs connected in series and parallel. **b** Capacitance and energy of the coaxial FSCs with different lengths. **c** A 1.5 m length of coaxial FSC; photograph of a sports watch powered by a textile bracelet composed of two coaxial FSCs; the letters “CAS” consisting of 44 LED array lighted by six FSCs; a smart bracelet charged by four FSCs connected in series

To widen the potential windows and improve the energy density of the coaxial Zn//Ti_3_C_2_T_*x*_ FSCs, two coaxial FSCs assembled in series and parallel were designed, respectively, as shown in Fig. 5a. The CV curves at the same scan rate showed the double voltage window and current when two coaxial FSCs connected in series and parallel, demonstrating the output voltage and capacitance can be adjusted easily by connecting multiple coaxial FSCs in series and parallel; thus, the fabricated FSCs could meet the energy and power requirements of energy storage devices in practical applications. The capacitance and energy of coaxial FSCs also increased proportionally with the increase in device lengths (Fig. 5b), so it is essential to realize the continuous fabrication of the FSC in practical application. From Fig. 5c, it can be seen that a length of 1.5 m coaxial FSC could be assembled at the same time because of the successful preparation of the meters of Zn fiber anode and Ti_3_C_2_T_*x*_ cathode. The softness of the coaxial FSC makes it possible to weave a fabric, so we wove a wristband by employing two coaxial FSCs, which were then used to power a sports watch. Furthermore, six coaxial FSCs were embedded into a glove to drive an LED array, and a smart bracelet could be charged by the plain fabric woven using coaxial FSCs.

## Conclusions

The high-performance Zn-ion FSC was obtained by designing the braided coaxial structures and utilization of Ti_3_C_2_T_*x*_ MXene cathode, which exhibited a high areal capacitance of 214 mF cm^**−**2^ and energy density of 42.8 μWh cm^**−**2^ at 5 mV s^**−1**^. Moreover, the FSC showed excellent cycling stability and good capacitance retention at bending, kitting, and braiding states. The letters “CAS” composed of 44 LED arrays could be lighted by six FSCs woven into textiles. This work represents a step toward the mass production of wearable energy storage devices and the potential for use in practical applications.

## Supplementary Information

Below is the link to the electronic supplementary material.

Supplementary file1 (PDF 819 kb)
