# Supplementary material for: Continuous Fabrication of Ti3C2Tx MXene-Based Braided Coaxial Zinc-Ion Hybrid Supercapacitors with Improved Performance
Source: Nanomicro Lett. 2021 Dec 15;14:34. doi: 10.1007/s40820-021-00757-6 (PMC8671578; doi:10.1007/s40820-021-00757-6)
Supplement: Supplementary file 1 — Supplementary file1 (PDF 819 kb) [file 40820_2021_757_MOESM1_ESM.pdf]

Supporting Information for

Continuously Fabrication of $Ti_3C_2T_x$ MXene-Based Braided Coaxial Zinc-Ion Hybrid Supercapacitors with Improved Performance

Bao Shi[1, +], La Li[2, +], Aibing Chen[1, *], Tien-Chien Jen[3], Xinying Liu[4], Guozhen Shen[2, *]

[1]Hebei University of Science and Technology, 70 Yuhua Road, Shijiazhuang 050018, P. R. China

[2]State Key Laboratory for Superlattices and Microstructures Institute of Semiconductors Chinese Academy of Sciences & Center of Materials Science and Optoelectronic Engineering, University of Chinese Academy of Sciences, Beijing 100083, P. R. China

[3]Department of Mechanical Engineering Science, Kingsway Campus, Auckland Park, University of Johannesburg, Johannesburg. 2006, South Africa

[4]Institute for Development of Energy for African Sustainability, University of South Africa, Private Bag X6, Florida 1710, South Africa

[+]B. Shi and L. Li contributed equally to this work

*Corresponding authors. E-mail: chen_ab@163.com (Aibing Chen), gzshen@semi.ac.cn (Guozhen Shen)

Supplementary Figures and Table

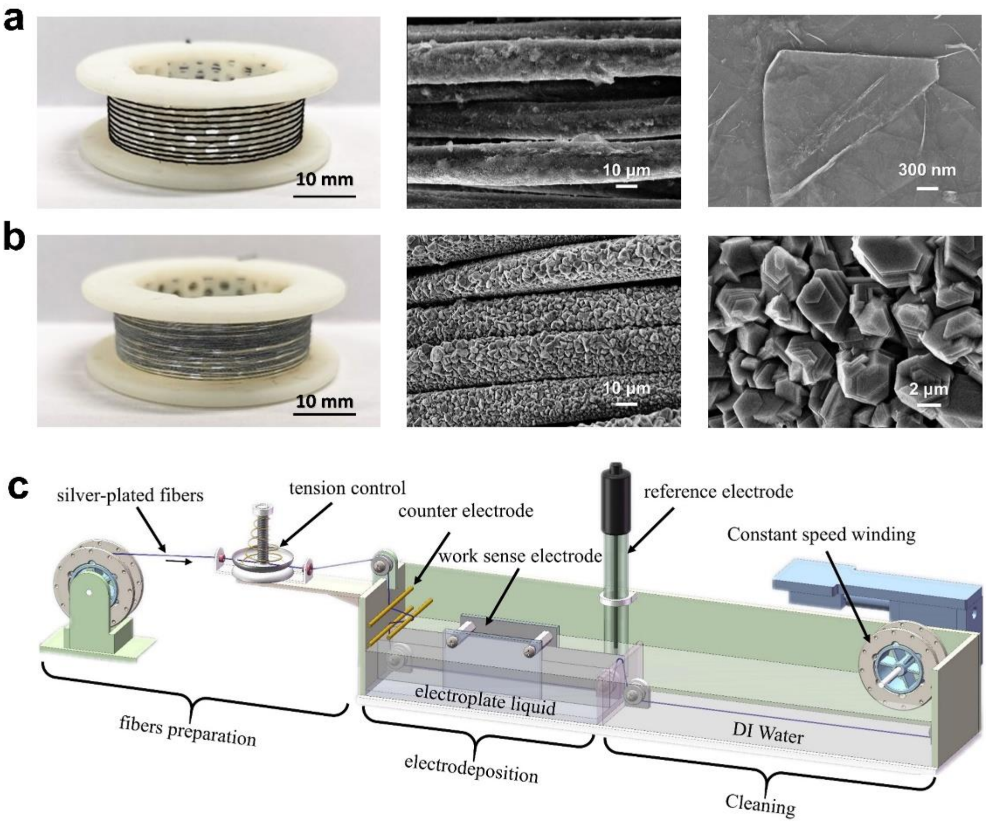

Fig. S1 Preparation of $Ti_3C_2T_x$ MXene and Zinc coated yarns. **a** 1.5 m of the $Ti_3C_2T_x$ MXene fiber cathode. **b** Meters of Zinc-coated yarn. **c** Structure drawing of self-made electrodeposition equipment

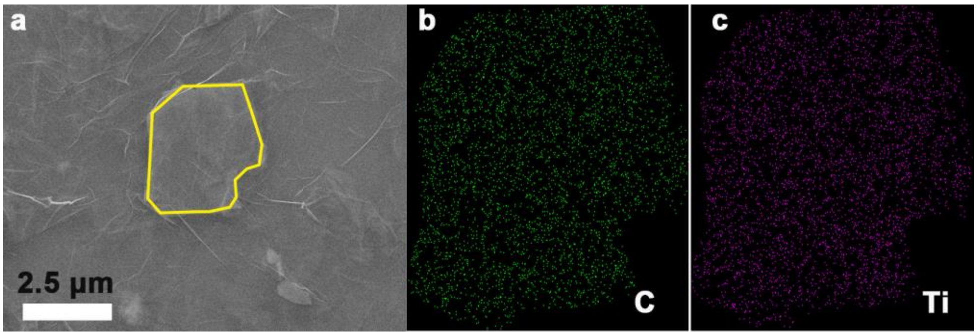

Fig. S2 The mapping images of the $Ti_3C_2T_x$ MXene cathode. **a** SEM images of the $Ti_3C_2T_x$. **b** C element. **c** Ti element

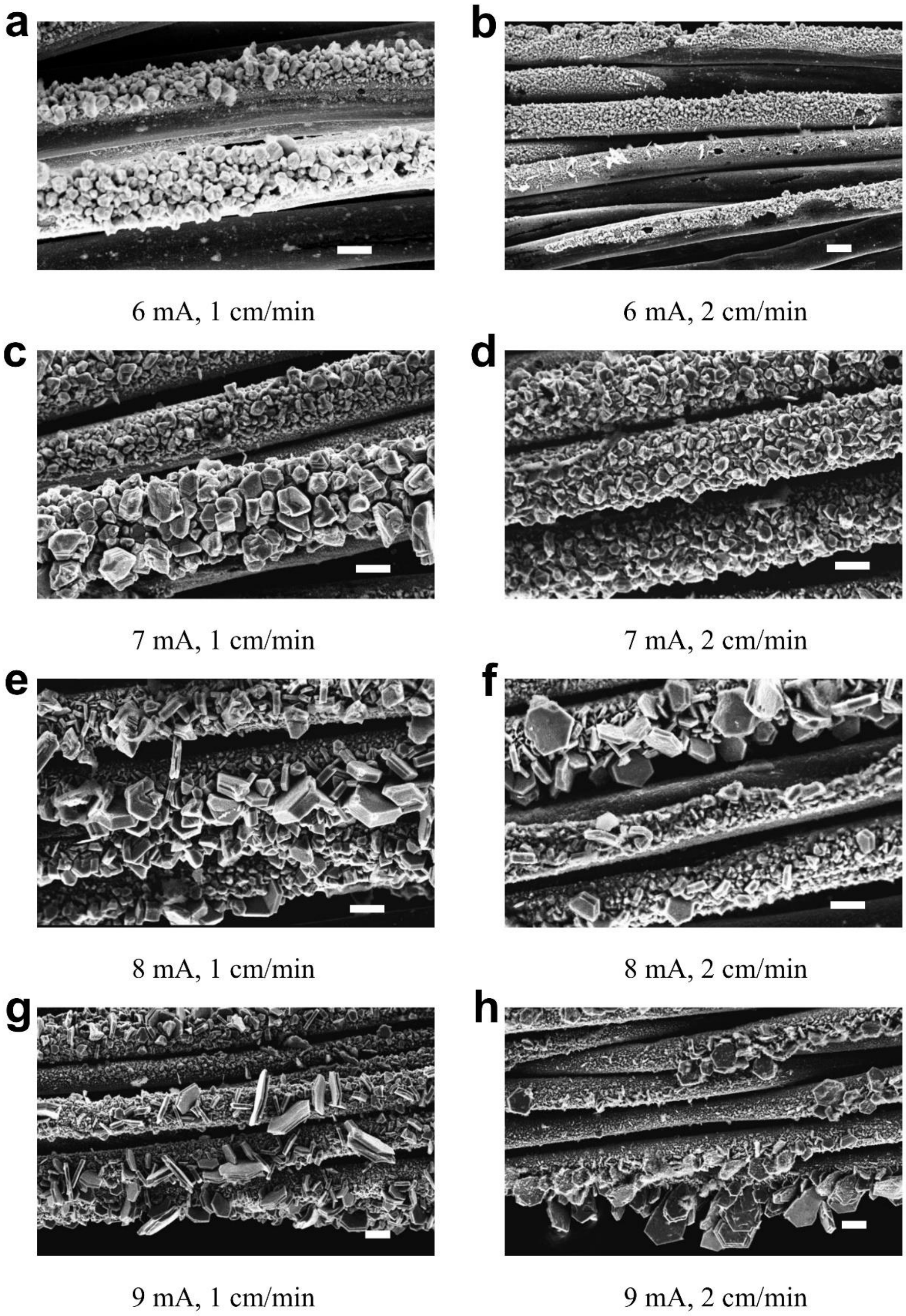

Fig. S3 SEM images of Zinc-coated fibers at different currents and speeds. scale bars: 10 μm

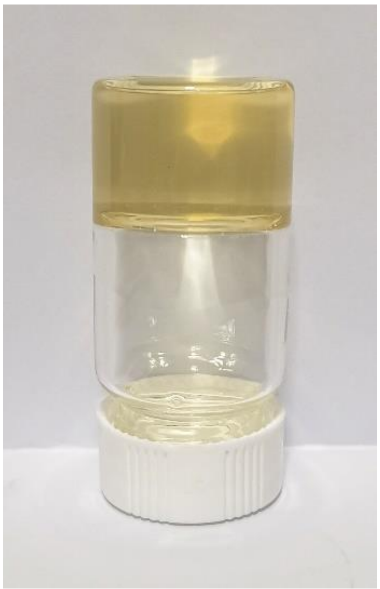

Fig. S4 Picture of the solid-state electrolyte of gelatin with $ZnSO_4$

Table S1 Simulated parameters of the coaxial FSCs with braided and winded coaxial structures

Parameters	Braided coaxial structure	Winded
Diameter of inner electrode (mm)	0.4	0.4
Diameter of external yarn (mm)	0.16	0.16
Diameter of braiding or winding (mm)	0.6	0.6
Pitch of braiding or winding (mm)	0.162	0.48
Number of external yarns	12(6 yarns left-handed, 6 yarns right-handed)	1(Parallel winding)
Voltage of inner electrode (V)	0.6	0.6
Voltage of external electrode (V)	-0.6	-0.6
Volume ratio of external yarns	1：1	

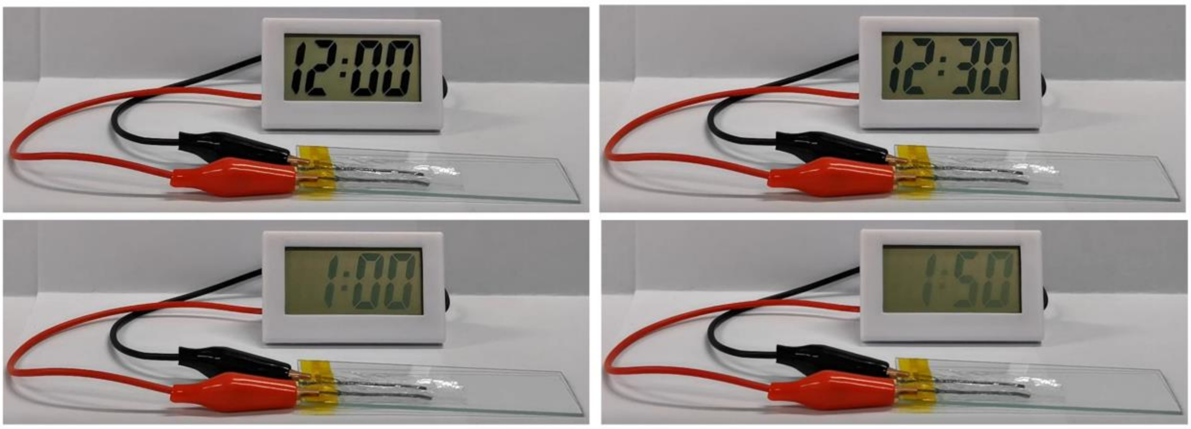

Fig. S5 Two coaxial FSCs in series power the electronic meter for 110 min
